# *Crataegus pinnatifida*: Chemical Constituents, Pharmacology, and Potential Applications

**DOI:** 10.3390/molecules19021685

**Published:** 2014-01-30

**Authors:** Jiaqi Wu, Wei Peng, Rongxin Qin, Hong Zhou

**Affiliations:** Department of Pharmacology, College of Pharmacy, The Third Military Medical University, Chongqing 400038, China

**Keywords:** *Crataegus pinnatifida*, chemical composition, pharmacology, toxicology

## Abstract

*Crataegus pinnatifida* (Hawthorn) is widely distributed in China and has a long history of use as a traditional medicine. The fruit of *C. pinnatifida* has been used for the treatment of cardiodynia, hernia, dyspepsia, postpartum blood stasis, and hemafecia and thus increasing interest in this plant has emerged in recent years. Between 1966 and 2013, numerous articles have been published on the chemical constituents, pharmacology or pharmacologic effects and toxicology of *C. pinnatifida*. To review the pharmacologic advances and to discuss the potential perspective for future investigation, we have summarized the main literature findings of these publications. So far, over 150 compounds including flavonoids, triterpenoids, steroids, monoterpenoids, sesquiterpenoids, lignans, hydroxycinnamic acids, organic acids and nitrogen-containing compounds have been isolated and identified from *C. pinnatifida*. It has been found that these constituents and extracts of *C. pinnatifida* have broad pharmacological effects with low toxicity on, for example, the cardiovascular, digestive, and endocrine systems, and pathogenic microorganisms, supporting the view that *C. pinnatifida* has favorable therapeutic effects. Thus, although *C. pinnatifida* has already been widely used as pharmacological therapy, due to its various active compounds, further research is warranted to develop new drugs.

## 1. Introduction

*Crataegus pinnatifida*, including *Crataegus pinnatifida* Bge. var. major N. E. Br. and *C. pinnatifida* Bge, is a traditional, popular Chinese medicinal herb that belongs to the Rosaceae family and is widely distributed in the north of China [1]. Modern investigations have demonstrated that *C. pinnatifida* has various pharmacological effects on, for example, the cardiovascular, digestive, and endocrine systems, as well as on pathogenic microorganisms [2]. To date, over 150 chemical constituents have been identified from this plant, including flavonoids, triterpenoids, steroids, lignans, organic acids, and nitrogen-containing compounds [3]. Due to its wide spectrum of biological and pharmacological effects, *C. pinnatifida* has a long history of use as a medicinal plant in China. In the *Compendium of Materia Medica* (Bencao Gangmu), a famous Traditional Chinese Medicine monograph, the earliest use of dried fruit of *C. pinnatifida* has been described as treatment for cardiodynia, hernia, dyspepsia, postpartum blood stasis, and hemafecia [4]. In addition to being used as a therapeutic medicine, the slightly sour fruit of *C. pinnatifida* is commonly used as a delicious daily food source in China [5].

In the present review we summarize the research advances on the chemical composition, pharmacology and toxicology of *C. pinnatifida*, which will be important for the development of new drugs and full utilization of *C. pinnatifida*. Additionally, we have discussed the potential perspective for future investigations of *C. pinnatifida*.

## 2. Chemical Composition

Research on the chemical components of *C. pinnatifida* started in the 1960s. Currently, over 150 compositions have been isolated and identified from *C. pinnatifida*, such as flavonoids, triterpenoids, steroids, monoterpenoids, sesquiterpenoids, lignans, organic acids and nitrogen-containing compounds. In this part, we describe the main chemical constituents of *C. pinnatifida* and their structures (Table 1).

**Table 1:** Chemical compounds isolated from Chinese Hawthorn.

Classification	No.	Chemical component	Part of Plant	Reference
Flavonoids	**1**	Apigenin	Leaves	[6]
	**2**	Luteolin	Leaves	[7]
	**3**	Orientin	Leaves	[8]
	**4**	Isoorientin	Leaves	[8]
	**5**	Vitexin	Flower	[9]
	**6**	Vitexin rhamnoside	Flower	[9]
	**7**	Isovitexin	Leaves	[10]
	**8**	Hyperoside	Leaves	[11]
	**9**	Pinnatifinoside A	Leaves	[12]
	**10**	Pinnatifinoside B	Leaves	[12]
	**11**	Pinnatifinoside C	Leaves	[12]
	**12**	Pinnatifinoside D	Leaves	[12]
	**13**	Pinnatifinoside I	Leaves	[12]
	**14**	3′′′, 4′′′-di-*O*-Acetyl-2′′-*O*-α-rhamuosylvitexin	Leaves	[13]
	**15**	Schaftoside	Leaves	[14]
	**16**	Isoschaftoside	Leaves	[14]
	**17**	Neoschaftoside	Leaves	[14]
	**18**	Neoisoschaftoside	Leaves	[14]
	**19**	Cratenacin	Leaves	[15]
	**20**	Acetylvitexin	Flower	[16]
	**21**	Crataequinone B	Leaves	[17]
	**22**	Kaempferol	Leaves	[18]
	**23**	Quercetin	Leaves	[11]
	**24**	Bioquercetin	Leaves	[9]
	**25**	Herbacetin	Leaves	[19]
	**26**	Santin	Leaves	[19]
	**27**	5-Hydroxyauranetin	Leaves	[19]
	**28**	Rutin	Leaves	[17]
	**29**	8-Methoxykaempferol	Flower	[9]
	**30**	Pinnatifidin	Flower	[20]
	**31**	Kaempferol 3-neohesperidoside	Leaves,Fruit	[21]
	**32**	8-Methoxykaempferol 3-neohesperidoside	Leaves,Fruit	[21]
	**33**	Naringenin-5,7-di-glucoside	Leaves	[22]
	**34**	Eriodictyol-5,3′-di-glucoside	Leaves	[22]
	**35**	(+)-Taxifolin	Leaves	[23]
	**36**	(+)-Taxifolin 3-*O*-arabinopyranoside 3-*O*-arabinopyranoside	Leaves	[23]
	**37**	(+)-Taxifolin 3-*O*-xylopyranoside	Leaves	[23]
	**38**	Crateside	Leaves	[24]
	**39**	(+)-Catechin	Leaves	[13]
	**40**	(−) *E*-picatechin	Leaves	[13]
	**41**	Leucocyanidin	Fruit	[25]
	**42**	Proanthocyanidin A_2_	Leaves, Flower	[26]
	**43**	Procyanidin B_2_	Leaves, Flower	[26]
	**44**	Procyanidin B_4_	Leaves, Flower	[26]
	**45**	Procyanidin B_5_	Leaves, Flower	[26]
	**46**	Procyanidin C_1_	Leaves, Flower	[26]
	**47**	Procyanidin D_1_	Leaves, Flower	[26]
	**48**	Epicatechin-(4β→6)-Epicatechin-(4β→8)- epicatechin	Leaves, Flower	[26]
	**49**	Epicatechin-(4β→8)- epicatechin-(4β→6)-epicatechin	Leaves, Flower	[26]
	**50**	Procyanidin E_1_	Leaves, Flower	[26]
Triterpenoids & Steroids	**51**	Ursolic acid	Fruit	[27]
	**52**	2α,3β,19α-trihydroxyl ursolic acid	Leaves	[28]
	**53**	Corosolic acid	Fruit	[29]
	**54**	Cuneataol	Fruit	[30]
	**55**	Cycloartenol	Stem, Leaves	[31]
	**56**	Uvaol	Fruit	[27]
	**57**	Oleanolic acid	Seeds	[32]
	**58**	Maslinic acid	Fruit	[29]
	**59**	Butyrospermol	Stem, Leaves	[31]
	**60**	24-Methylene-24-dihydrolanosterol	Stem, Leaves	[31]
	**61**	Betulin	Fruit	[27]
	**62**	18,19-seco,2α,3β-Dihydroxy-19-oxo-urs-11,13(18)-dien-28-oic acid	Leaves	[33]
	**63**	β-Sitosterol	Fruit	[34]
	**64**	β-Daucosterol	Fruit	[34]
	**65**	Stigmosterol	Fruit	[34]
	**66**	24-Methylene-24-dihydrolanosterol	Stem, Leaves	[31]
Monoterpenes & sesquiterpenes	**67**	3,9-Dihydroxymegastigma-5-ene	Leaves	[33]
	**68**	(3*S*,5*R*,6*R*,7*E*)-Megatsigmane-7-ene-3-hydroxy-5, 6-epoxy-9-*O*-β-d-glucopyranoside	Leaves	[33]
	**69**	(3*R*,5*S*,6*S*,7*E*,9*S*)-Megastigman-7-ene-3,5,6,9-tetrol 9-*O*-β-d-glucopyranoside	Leaves	[35]
	**70**	(6*S*,7*E*,9*R*)-6,9-Dihydroxy-4,7-megastigmadien-3-one 9-*O*-[β-d-xylopyranosyl-(1′′→6′)-β-d-glucopyranoside]	Leaves	[35]
	**71**	Linarionoside C	Leaves	[36]
	**72**	Linarionoside A	Leaves	[36]
	**73**	Linarionoside B	Leaves	[36]
	**74**	3β-d-Glucopyranosyloxy-β-ionone	Leaves	[36]
	**75**	Icariside B_6_	Leaves	[36]
	**76**	Pisumionoside	Leaves	[36]
	**77**	(3*S*,5*R*,6*R*,7*E*,9*R*)-3,6-Epoxy-7-megastigmen-5,9-diol-9-*O*-β-d-glucopyranoside	Leaves	[36]
	**78**	(6*S*,7*E*,9*R*)-Roseoside	Leaves	[36]
	**79**	(6*R*,9*R*)-3-Oxo-α-ionol-9-*O*-β-d-glucopyranoside	Leaves	[36]
	**80**	4-[4β-O-β-d-Xylopyranosyl-(1′′→6′)-β-d-glucopyranosyl-2,6,6-trimethyl-1-cyclohexen-1-yl]-butan-2-one	Leaves	[35]
	**81**	(3*S*,9*R*)-3,9-Dihydroxy-megastigman-5-ene 3-*O*-primeveroside	Leaves	[35]
	**82**	(3*R*,5*S*,6*S*,7*E*,9*S*)-Megastiman-7-ene-3,5,6,9-tetrol	Leaves	[35]
	**83**	1β,9α-Dihydroxyeudesm-3-en-5β,6α,7α,11α H-12,6-olide	Fruit	[37]
	**84**	(5*Z*)-6-[5-(2-Hydroxypropan-2-yl)-2-methyltetrahydrofuran-2-yl] -3-methylhexa-1,5-dien-3-ol	Leaves	[35]
	**85**	(5*Z*)-6-[5-(2-O-β-d-Glucopyranosyl-propan-2-yl)-2-methyl tetrahydrofur-an-2-yl]-3-methylhexa-1,5-dien-3-ol	Leaves	[35]
	**86**	5-Ethenyl-2-[2-O-β-d-glucopyranosyl-(1′′→6′)-β-d-glucopyranosyl-propan-2-yl]-5-methyltetrahydrofuran-2-ol	Leaves	[35]
	**87**	Gibberellic acid	Fruit	[38]
Lignans	**88**	(2,3-Dihydro-2-(4-*O*-β-d-glueopyranosyl-3-methoxy-Phenyl)-3-hydroxymethyl-5-(3-hydroxypropyl)-7-methoxybenzofuran)	Leaves	[39]
	**89**	Shanyenoside A	Leaves	[40]
	**90**	(7*S*,8*R*)-Urolignoside	Leaves	[36]
	**91**	(−)-2a-*O*-(β-d-Glucopyranosyl)- lyoniresinol	Leaves	[36]
	**92**	Tortoside A	Leaves	[36]
	**93**	Verbascoside	Leaves	[36]
	**94**	Acernikol-4′′-*O*-β- d-glucopyranoside	Leaves	[36]
	**95**	*erythro*-1-(4-*O*-β-d-Glucopyranosyl-3-methoxyphenyl)-2-[4-(3-hydroxypropyl)-2,6-dimethoxyphenoxy]-1,3-propanediol	Leaves	[36]
	**96**	(7*S*, 8*R*)-5-Methoxydihydrodehydrodiconiferyl alcohol 4-*O*-β- d-glucopyranoside	Leaves	[36]
	**97**	Pinnatifidanin C I	Seeds	[41]
	**98**	Pinnatifidanin C II	Seeds	[41]
	**99**	Pinnatifidanin C III	Seeds	[41]
	**100**	Pinnatifidanin C IV	Seeds	[41]
	**101**	Pinnatifidanin C V	Seeds	[41]
	**102**	Pinnatifidanin C VI	Seeds	[40]
	**103**	Pinnatifidanin C VII	Seeds	[41]
	**104**	Pinnatifidanin C VIII	Seeds	[41]
	**105**	Pinnatifidanin B I	Seeds	[42]
	**106**	Pinnatifidanin B II	Seeds	[42]
	**107**	Pinnatifidanin B III	Seeds	[42]
	**108**	Pinnatifidanin B IV	Seeds	[42]
	**109**	Pinnatifidanin B V	Seeds	[42]
	**110**	Pinnatifidanin B VI	Seeds	[42]
	**111**	Pinnatifidanin B VII	Seeds	[42]
	**112**	Pinnatifidanin B VIII	Seeds	[42]
	**113**	Pinnatifidanin B IX	Seeds	[42]
Hydroxycinnamic acids	**114**	Chlorogenic acid	Leaves	[19]
	**115**	β-Coumaric acid	Fruit	[38]
	**116**	Caffeic acid	Fruit	[38]
	**117**	Ferulic acid	Fruit	[38
Organic acids	**118**	Benzoic acid	Leaves	[33]
	**119**	(*p*-Hydroxyphenyl) benzoic acid	Seed	[43]
	**120**	Gallic acid	Seed	[43]
	**121**	Protocatechuic acid	Seed	[43]
	**122**	Anisic acid	Fruit	[38]
	**123**	Vanillic acid	Fruit	[38]
	**124**	Syringic acid	Fruit	[38]
	**125**	Gentisic acid	Fruit	[38]
	**126**	Malic acid	Fruit	[44]
	**127**	Citric acid	Fruit	[44]
	**128**	Quinic acid	Fruit	[44]
	**129**	Pyruvic acid	Fruit	[44]
	**130**	Tartaric acid	Fruit	[44]
	**131**	Succinic acid	Fruit	[34]
	**132**	Fumaric acid	Seed	[32]
	**133**	Ascorbic acid	Shoot	[45]
	**134**	2-(4-Hydroxy-2-benzyl) malic acid	Seed	[32]
	**135**	Palmitic acid	Fruit	[46]
	**136**	Stearic acid	Fruit	[46]
	**137**	Oleic acid	Fruit	[46]
	**138**	Linoleic acid	Fruit	[46]
Nitrogenous compounds	**139**	Isobutylamine	Leaves	[47]
	**140**	Ethylamine	Leaves	[47]
	**141**	Dimethylamine	Leaves	[47]
	**142**	Trimethylamine	Leaves	[47]
	**143**	Isoamyl amine	Leaves	[47]
	**144**	Ethanolamine	Leaves	[47]
	**145**	Choline	Leaves	[47]
	**146**	Acetylcholine	Leaves	[47]
	**147**	Spermindine	Leaves	[47]
	**148**	*O*-Methoxyphenethylamine	Leaves	[47]
	**149**	Tyramine	Leaves	[47]
	**150**	Phenylethylamine	Leaves	[47]
*Other compounds*	**151**	Hentriacontane	Fruit	[27]
	**152**	Hexadecanoic acid, octaconsyl ester	Fruit	[27]
	**153**	Eicosanoic acid, octatriacontyl ester	Fruit	[27]
	**154**	Nonacosan-10-ol	Fruit	[27]
	**155**	2,8-Dihydroxy-3,4,7-trimethoxydibenzofuran	Bark, sapwood	[48]
	**156**	(*Z*)-3-hexenyl-*O*-β-d-glucopyranosyl-(1′′→6′)-β-d-glucopyranoside	Leaves	[35]
	**157**	(*Z*)-3-Hexenyl-*O*-β-d-xylopyranosyl-(1′′→6′)-β-d-glucopyranoside	Leaves	[35]
	**158**	(*Z*)-3-Hexenyl-*O*-β-d-rhamnopyranosyl-(1′′→6′)-β-d-glucopyranoside	Leaves	[35]

### 2.1. Flavonoids

**Figure 1:**
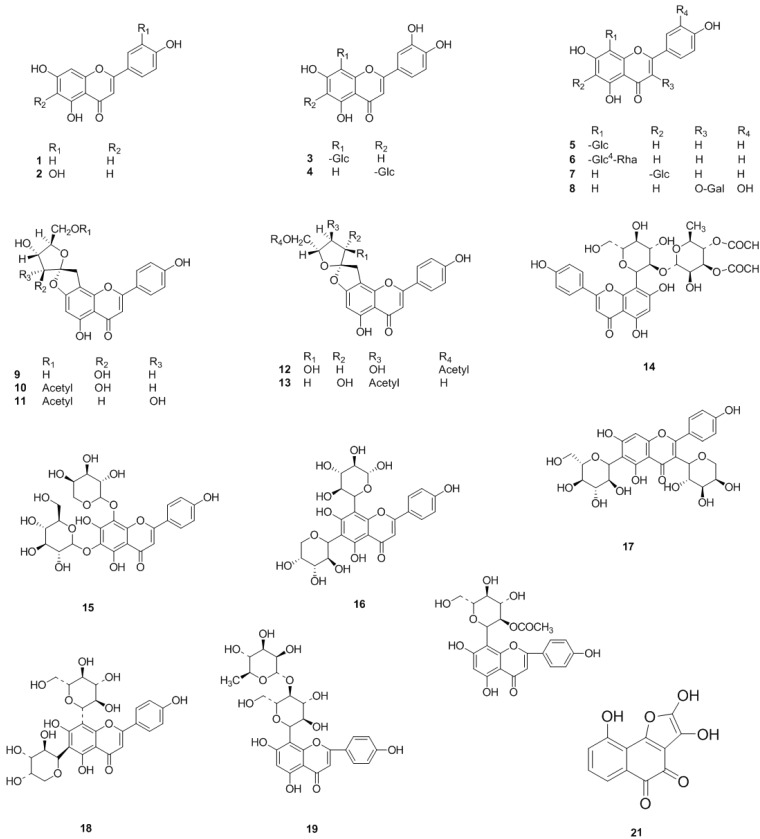
Chemical structures of flavones in *C. pinnatifida*.

**Figure 2:**
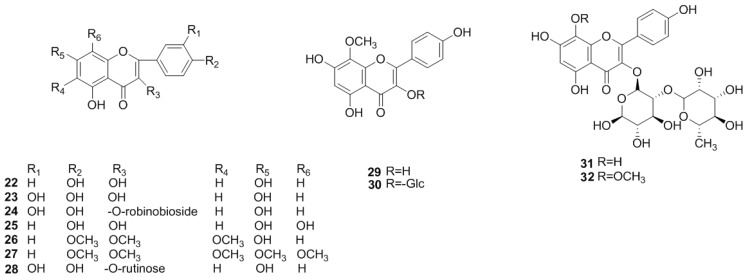
Chemical structures of flavonols in *C. pinnatifida*.

**Figure 3:**
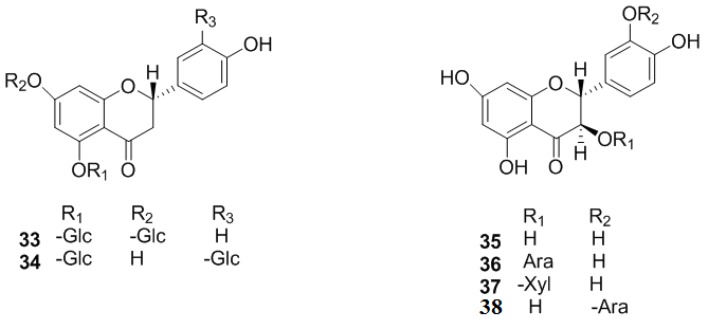
Chemical structures of flavanones and flavanonols.

**Figure 4:**
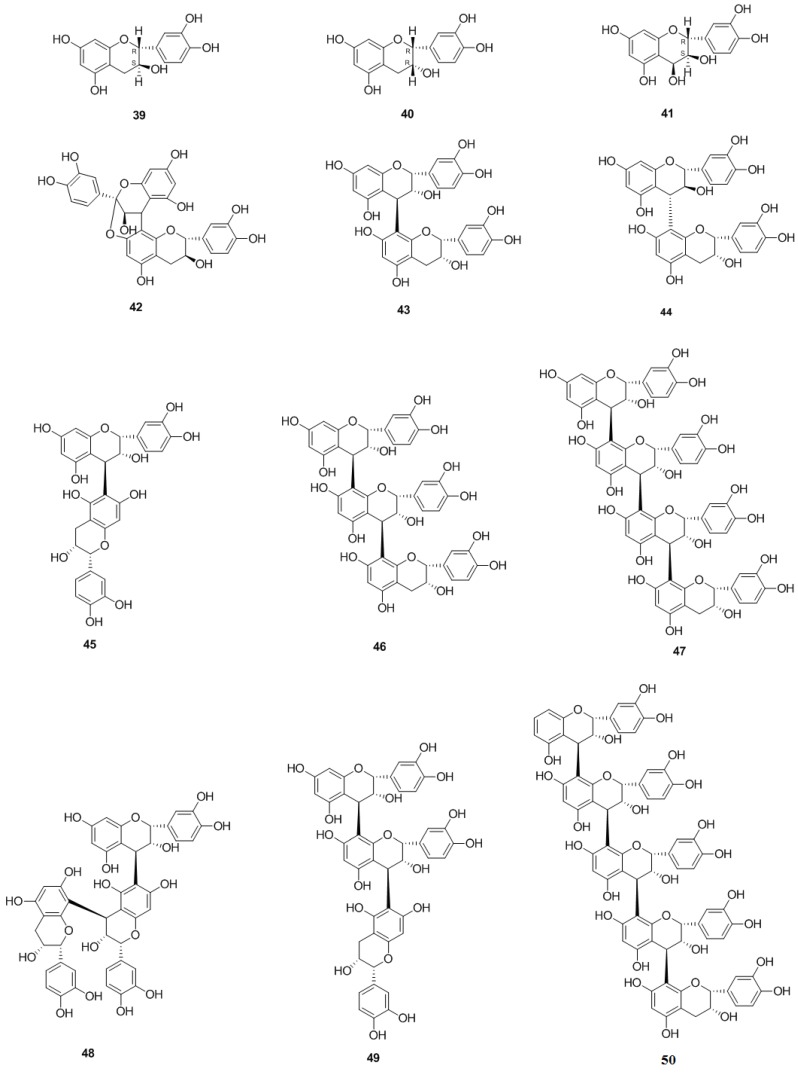
Chemical structures of flavanols and the polymers of flavanols in *C. pinnatifida*.

Flavonoids and its derivatives, including flavones, flavonols, flavanones, flavanonols, flavanols and polymers of flavanols, are the most abundant chemical components of *C. pinnatifida* (Figure 1, Figure 2, Figure 3 and Figure 4).

#### 2.1.1. Flavones

Since the first study of *C. pinnatifida* in the 1960s, flavones have been isolated and identified from the leaves and flowers of *C. pinnatifida*. These flavones are a series of compounds whose aglycones are apigenin or luteolin. These flavones include apigenin (**1**), luteolin (**2**), orientin (**3**), iso-orientin (**4**), vitexin (**5**), vitexin rhamnoside (**6**), isovitexin (**7**), hyperoside (**8**), pinnatifinoside A–D, I (**9**–**13**), 3′′′,4′′′-di-*O*-acetyl-2′′-*O*-α-rhamnosylvitexin (**14**), schaftoside (**15**), isoschaftoside (**16**), neoschaftoside (**17**), neoisoschaftoside (**18**), cratenacin (**19**), acetylvitexin (**20**). Additionally, crataequinone B (**21**) was isolated from the leaves of *C. pinnatifida* [17] (Figure 1).

#### 2.1.2. Flavonols

Flavonols are also as abundant in *C. pinnatifida* as flavones. The configuration of flavonols of *C. pinnatifida* is mainly quercetin-and kaempferol-like, including kaempferol (**22**), quercetin (**23**), bioquercetin (**24**), herbacetin (**25**), santin (**26**), 5-hydroxyauranetin (**27**), rutin (**28**), 8-methoxy-kaempferol (**29**), pinnatifidin (**30**), kaempferol 3-neohesperidoside (**31**), and 8-methoxykaempferol 3-neohesperidoside (**32**) (Figure 2).

#### 2.1.3. Flavanones and Flavanonols

In 1971, naringenin 5,7-diglucoside (**33**) and eriodictyol-5,3′-diglucoside (**34**) were isolated from the leaves of *C. pinnatifida* [22]. Later, [(+)-taxifolin (**35**)], [(+)-taxifolin-3-*O*-arabinopyranoside (**36**)] and [(+)-taxifolin 3-*O*-xylopyranoside (**37**)] were also isolated from the leaves [23]. Additionally, crateside (**38**) was isolated [24] (Figure 3).

#### 2.1.4. Flavanols and the Polymers of Flavanols

Flavanols and flavanol polymers, the elementary units of which were (+)-catechin (**39**)], (‒)-*E*-picatechin (**40**) and leucocyanidin (**41**), are also abundant in *C. pinnatifida* [13,25]. The polymers are a series of compounds where these three compounds are polymerized [26]. Dimers include proanthocyanidin A2 (**42**), procyanidin B2, B4, B5 (**43**–**45**), and trimers include procyanidin C1 (**46**), procyanidin D1 (**47**), epicatechin-(4β-6)-epicatechin-(4β-8)-epicatechin (**48**), epicatechin-(4β-8)-epi-catechin-(4β-6)-epicatechin (**49**) and procyanidin E1 (**50**) (Figure 4). Dimers and trimers were isolated and identified from *C. pinnatifida* in 2002.

### 2.2. Triterpenoids and Steroids

#### 2.2.1. Triterpenoids

**Figure 5:**
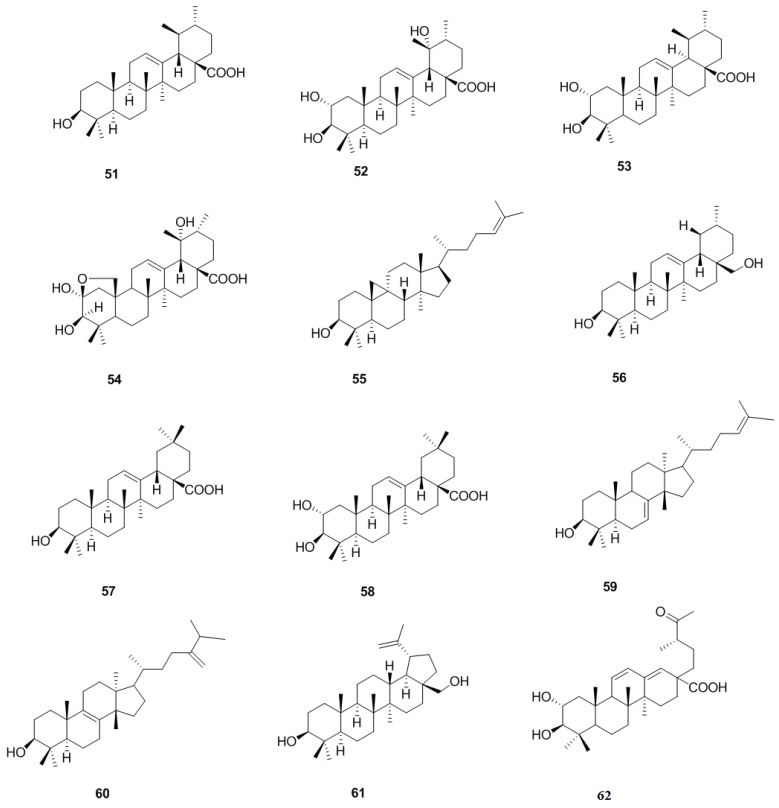
Chemical structures of triterpenoids in *C. pinnatifida*.

Since the first study of *C. pinnatifida* in the 1960s, triterpenoids and their derivatives have been isolated and identified. These triterpenoids are classified into tetracyclic triterpenoids and pentacyclic triterpenoids, such as ursolic acid (**51**), 2α,3β,19α-trihydroxyursolic acid (**52**), corosolic acid (**53**), cuneataol (**54**), cycloartenol (**55**), uvaol (**56**), oleanolic acid (**57**), crataegolic acid (**58**), butyrospermol (**59**), 24-methylene-24-dihydrolanosterol (**60**), betulin (**61**), and 18,19-seco-2α,3β-dihydroxy-19-oxo-urs-11,13(18)-dien-28-oic acid (**62**) [27,28,29,30,31,32,33] (Figure 5).

#### 2.2.2. Steroids

**Figure 6:**
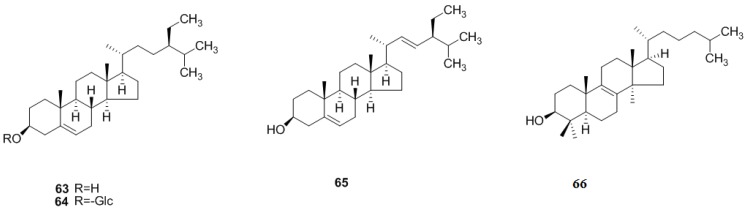
Chemical structures of steroids in *C. pinnatifida*.

So far, four steroids were isolated from *C. pinnatifida*. In 1997, 24-methylen-24-dihydrolanosterol (**66**) was isolated from stems and leaves of *C. pinnatifida* [31]. Later, β-sitosterol (**63**), β-daucosterol (**64**), stigmosterol (**65**) were isolated from fruits of *C. pinnatifida* [34] (Figure 6).

### 2.3. Monoterpenoids and Sesquiterpenoids

**Figure 7:**
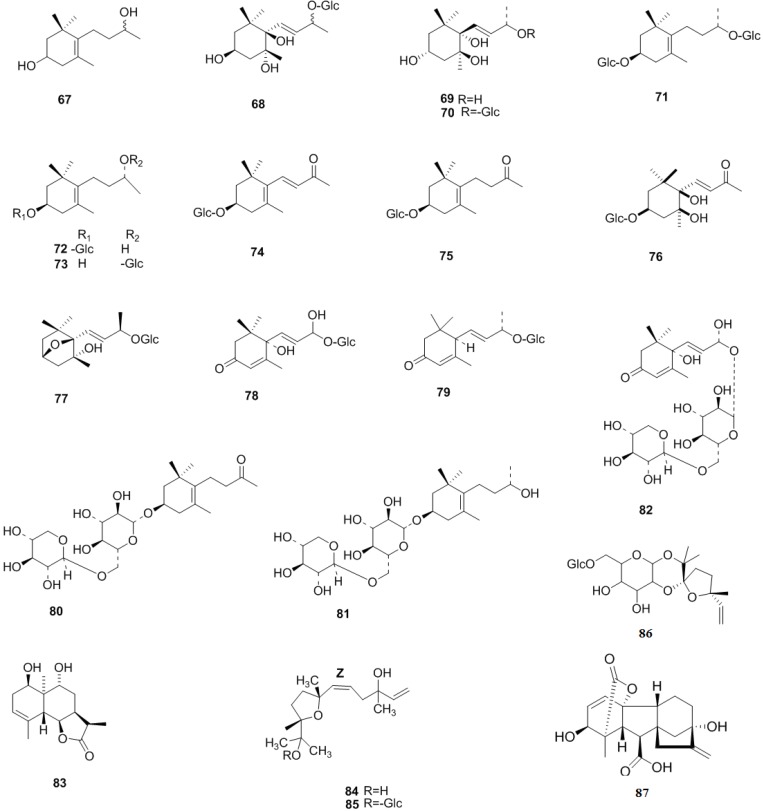
Chemical structures of monoterpenoids and sesquiterpenoids in *C. pinnatifida*.

Monoterpenoids and sesquiterpenoids are the main constituents of the volatile oil from *C. pinnatifida*, which is an important raw material in the spice and medical industry. Monoterpenoids and sesquiterpenoids are also abundant in the leaves of *C. pinnatifida*. 3,9-Dihydroxymegastigma-5-ene (**67**) and (3*S*,5*R*,6*R*,7*E*)-megatsigmane-7-ene-3-hydroxy-5,6-epoxy-9-*O*-β-d-glucopyranoside (**68**) were new compounds firstly isolated from the leaves of *C. pinnatifida* and identified in 2010 [33]. Later, (3*R*,5*S*,6*S*,7*E*,9*S*)-megastigman-7-ene-3,5,6,9-tetrol-9-*O*-β-d-glucopyranoside (**69**) and (6*S*,7*E*, 9*R*)-6,9-dihydroxy-4,7-megastigmadien-3-one 9-*O*-[β-d-xylopyranosyl-(1′′→6′)-β-d-glucopyranoside] (**70**) were isolated and identified from *C. pinnatifida* leaves in 2011 [35]. Additionally, linarionoside C (**71**), linarionoside A, B (**72**–**73**), 3β-glucopyranosyloxy-β-ionone (**74**), icariside B6 (**75**), pisumionoside (**76**), (3*S*,5*R*,6*R*,7*E*,9*R*)-3,6-epoxy-7-megastigmen-5,9-diol-9-*O*-β-d-glucopyranoside (**77**), (6*S*,7*E*,9*R*)-roseoside (**78**) and (6*R*,9*R*)-3-oxo-α-ionol-9-*O*-β-d-glucopyranoside (**79**) have been isolated from the leaves of *C. pinnatifida* in 2010 [36] (Figure 7).

What’s more, 1β,9α-dihydroxyeudesm-3-en-5β,6α,7α,11α*H*-12,6-olide (**83**) was isolated and identified from the fruits of *C. pinnatifida* [37]. In addition, six others were subsequently isolated from the leaves of *C. pinnatifida*, including 4-[4β-O-β-d-xylopyranosyl-(1′′→6′)- β-d-glucopyranosyl-2,6,6-trimethyl-1-cyclohexen-1-yl]-butan-2-one (**80**), (3*S*,9*R*)-3,9-dihydroxymegastigman-5-ene 3-*O*-primeveroside (**81**), (3*R*,5*S*,6*S*,7*E*,9*S*)-megastiman-7-ene-3,5,6,9-tetrol (**82**), (5*Z*)-6-[5-(2-hydroxypropan-2-yl)-2-methyltetrahydrofuran-2-yl]-3-methylhexa-1,5-dien-3-ol (**84**), (5*Z*)-6-[5-(2-O-β-d-glucopyranosyl-propan-2-yl)-2-methyltetrahydrofuran-2-yl]-3-methylhexa-1,5-dien-3-ol (**85**), 5-ethenyl-2-[2-O-β-d-glucopyranosyl-(1′′→6′)-β-d-glucopyranosyl-propan-2-yl]-5-methyltetrahydrofuran-2-ol (86) [35], and gibberellic acid (87) [38] (Figure 7).

### 2.4. Lignans

**Figure 8:**
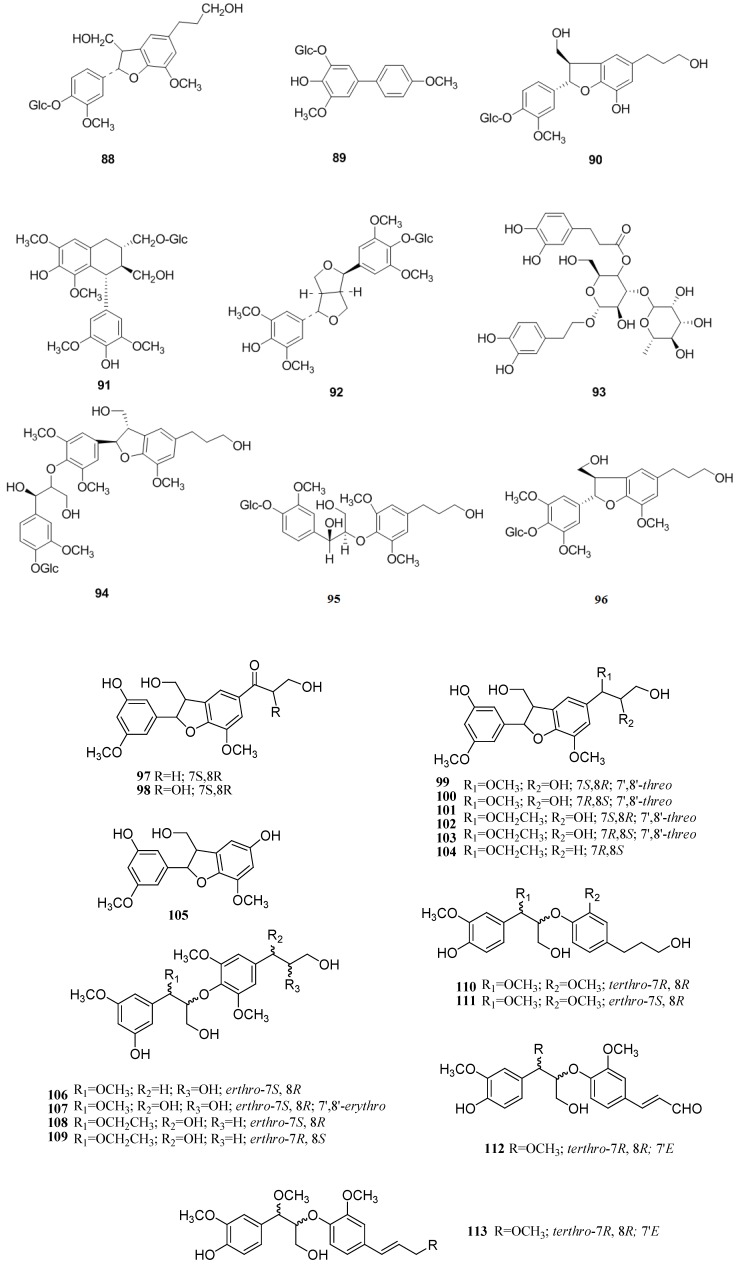
Chemical structures of lignans in *C. pinnatifida*.

Lignans, a kind of natural product containing two phenylpropane frameworks, is another characteristic component of *C. pinnatifida*, where they mainly exist in the leaves. A new identified compound, shanyenoside (A) (**89**), was isolated from the leaves of *C. pinnatifida* in 2006 [40]. In 2009, (2,3-dihydro-2-(4-*O*-β-d-glueopyranosyl-3-methoxyphenyl)-3-hydroxymethyl-5-(3-hydroxypropyl)-7-methoxybenzofuran) (**88**) was isolated from the leaves of *C. pinnatifida* [39]. Later, (7*S*,8*R*)-urolignoside (**90**), (−)-2a-*O*-(β-d-glucopyranosyl)lyoniresinol (**91**), tortoside A (**92**), verbascoside (**93**), acernikol-4′′-*O*-β-d-glucopyranoside (**94**), erythro-1-(4-*O*-β-d-glucopyranosyl-3-methoxyphenyl)-2-[4-(3-hydroxypropyl)-2,6-dimethoxyphenoxy]-1,3-propanediol (**95**) and (7*S*,8*R*)-5-methoxydihydro-dehydrodiconiferyl alcohol 4-*O*-β-d-glucopyranoside (**96**) were also isolated from the leaves of *C. pinnatifida* in 2010 [36]. Recently, some novel neolignans were isolated from the seeds of *C. pinnatifida*, including pinnatifidanin C I–VIII, pinnatifidanin B I–IX (**97**–**112**) [40,41,42] (Figure 8).

### 2.5. Hydroxycinnamic Acids

**Figure 9:**
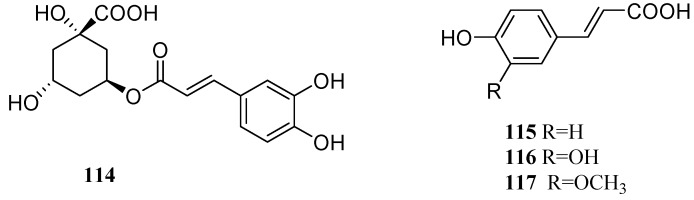
Chemical structures of hydroxycinnamic acids in *C. pinnatifida*.

Lots of hydroxycinnamic acids were also isolated from the leaves and fruits of *C. pinnatifida*, including chlorogenic acid (**114**), β-coumaric acid (**115**), caffeic acid (**116**), and ferulic acid (**117**) [19,38] (Figure 9).

### 2.6. Organic Acids

**Figure 10:**
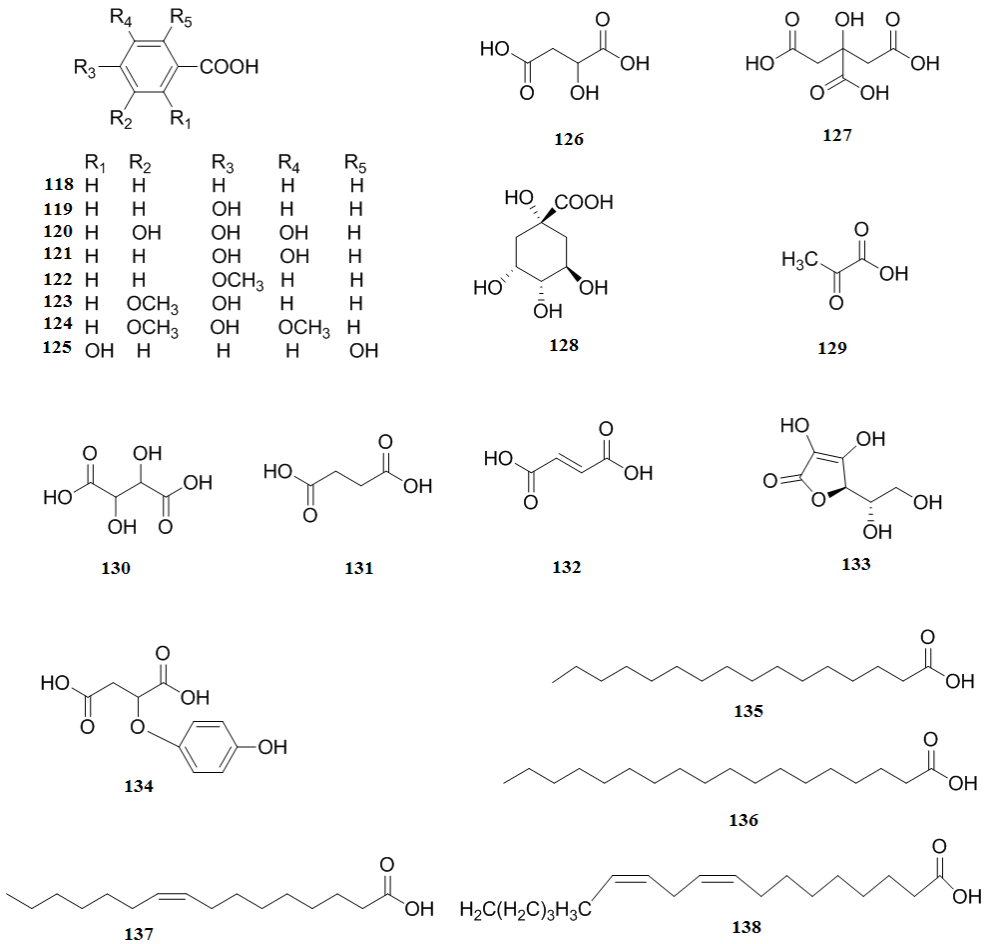
Chemical structures of organic acids in *C. pinnatifida*.

Organic acids of *C. pinnatifida* mainly include phenolic acids and other organic acids. Phenolic acids include benzoic acid (**118**), (*p*-hydroxyphenyl)benzoic acid (**119**), gallic acid (**120**), protocatechuic acid (**121**), anisic acid (**122**), vanillic acid (**123**), syringic acid (**124**), gentisic acid (**125**) [19,33,40,41]. Other organic acids include malic acid (**126**), citric acid (**127**), quinic acid (**128**), pyruvic acid (**129**), tartaric acid (**130**), succinic acid (**131**), fumaric acid (**132**), ascorbic acid (**133**), 2-(4-hydroxy2 benzyl) malic acid (**134**), palmitic acid (**135**), stearic acid (**136**), oleic acid (**137**), and linoleic acid (**138**) [32,34,38,43,44,45,46] (Figure 10).

### 2.7. Nitrogen-containing Compounds

**Figure 11:**
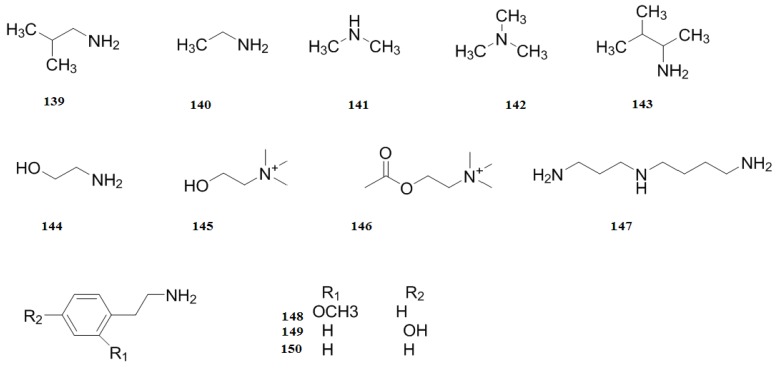
Chemical structures of nitrogen-containing compounds in *C. pinnatifida*.

So far, twelve nitrogen-containing compounds were isolated from the leaves of *C. pinnatifida*. In 1990, the nitrogen-containing compounds isobutylamine (**139**), ethylamine (**140**), dimethylamine (**141**), trimethylamine (**142**), isoamylamine (**143**), ethanolamine (**1>44**), choline (**145**), acetylcholine (**146**), spermindine (**147**), *O*-methoxyphenethylamine (**148**), tyramine (**149**), and phenylethylamine (**150**) were isolated and identified [47] (Figure 11).

### 2.8. Others

**Figure 12:**
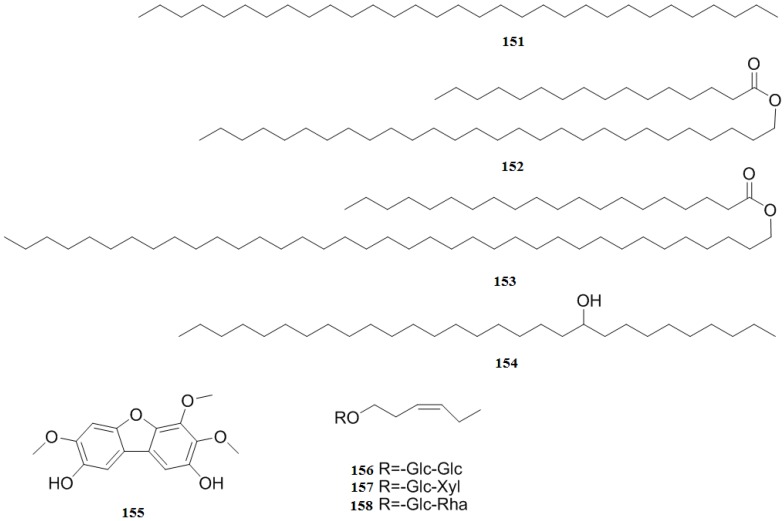
Chemical structures of other compounds in *C. pinnatifida*.

A compound identified as 2,8-dihydroxy-3,4,7-trimethoxydibenzofuran (**151**) was isolated from the bark and sapwood of *C. pinnatifida* [48]. Hentriacontane (**152**), (hexadecanoic acid, octaconsyl ester) (**153**), (eicosanoic acid, octatriacontyl ester) (**154**) and nonacosan-10-ol (**155**) were also isolated from the fruits of *C. pinnatifida* [27]. Recently, three new compounds, (*Z*)-3-hexenyl-*O*-β-d-gluco-pyranosyl-(1′′→6′)-β-d-glucopyranoside (**156**), (*Z*)-3-hexenyl-*O*-β-d-xylopyranosyl-(1′′→6′)-β-d-glucopyranoside (**157**), and (*Z*)-3-hexenyl-*O*-β-d-rhamnopyranosyl-(1′′→6′)-β-D-glucopyranoside (**158**) were isolated from the leaves of *C. pinnatifida* [35] (Figure 12). Additionally, plenty of sugars and sugar alcohols were found in the *C. pinnatifida,* including glucose, sucrose, fructose, sorbitol, and myoinositol, with fructose being the most abundant sugar in the fruits [49].

## 3. Biological Properties

### 3.1. Cardiovascular System Effects

#### 3.1.1. Lipid Regulating and Anti-atherosclerosis Effects

Total flavonoids of the leaves from *C. pinnatifida* obviously decreased the serum levels of total cholesterol (TC) and triglyceride (TG) through gavage in high-fat/cholesterol rabbit models [50]. Total flavonoids of *C. pinnatifida* at a middle concentration of 50 µg/mL were able to promote the proliferation of preadipocytes but inhibit its differentiation. In addition, total flavonoids of *C. pinnatifida* inhibit mature adipocyte secretion of leptin and PAI-1 in a dose-dependent manner [51]. Moreover, total flavonoids of *C. pinnatifida* can markedly decrease the levels of total cholesterol (TC) and triglyceride (TG) in serum by controlling the gene expressions including FAS, HSL, TGH, SREBP-1c [52].

Atherosclerosis is a risk factor for coronary disease. There are a lot of theories about the pathogenesis of atherosclerosis, one of which is abnormal cholesterol levels [53]. The flavone extracted from *C. pinnatifida* by 70% ethanol obviously decreased the serum levels of total cholesterol (TC), triglyceride and low-density lipoprotein cholesterol (LDLC) in high-fat/cholesterol rabbit and rat models, suggesting its use to treat atherosclerosis [54].

Investigations have shown that the main antihyperlipidemic effect constituents of *C. pinnatifida* are hyperin and ursolic acid. Two animal models of hyperlipidemia were established in mice with 75% yolk and Triton-WR 1339 400 mg/kg (ip), respectively. The animals were administrated with hyperoside or ursolic acid extracted from *C. pinnatifida* in two doses. The total cholestrol (TCH), triglyceride (TG), high density lipoprotein (HDL) and superoxide dismutase (SOD) activities in serum were measured. In comparison with control groups, TCH levels in all the dosed groups were significantly decreased, while HDL and SOD activity increased; the ratio of total cholesterol/high-density lipoprotein (TC/HDL) was reduced, too. This effect could lessen damages to vascular endothelium induced by oxygen free radical (OFR) in hyperlipoidemia, thus preventing atherosclerosis [55,56]. Total flavonoids of *C. pinnatifida* had significant antihyperlipidemic effects and enhanced the vascular function of hyperlipidemia model rats, the mechanism of which might be relevant to the increased levels of nitric oxide (NO) in the serum and the reduction in endothelin (ET) synthesis [57,58].

#### 3.1.2. Resistance to Chronic Heart Failure

In many clinical trials, *C. pinnatifida* extract was confirmed to be effective in the treatment of patients with chronic heart failure defined as NYHA functional class II. There were no severe side effects observed [59,60,61]. In another clinical trial, *C. pinnatifida* extract WS 1442, a dry extract from hawthorn leaves with flowers (dry extract ratio = 4–6.6:1, extraction solvent: ethanol 45%) standardized to 18.75% oligomeric procyanidines (OPC), was also effective in the treatment of patients with chronic stable New York Heart Association class-III heart failure. The most important constituents for the therapeutic effects of WS 1442 are OPC [62]. *C. pinnatifida* was capable of regulating and ameliorating cardiovascular system effects, as it enhanced myocardial contractility and expanded the coronary artery, lessened heart rhythm, myocardial oxygen consumption and peripheral resistance [63]. The mechanism of coronary artery expansion was relevant to the β-adrenergic receptor. The flavonoids of *C. pinnatifida* might be a new alternative botanical drug for chronic heart failure because of its good test results in pharmacological experiments.

#### 3.1.3. Antihypertensive Effects

The extracts of *C. pinnatifida* could reduce blood pressure slowly and enduringly in mice, rabbits and cats, the mechanism of which was related to expanded peripheral vessels, and the active components were the flavanol dimers or multimers [64,65]. On compounding hypertension and hyperlipoidemia rats, extracts of *C. pinnatifida* at the doses of 1.5 and 2.25 g/kg/d could maintain rats’ blood pressure [66].

#### 3.1.4. Anti-myocardial Ischemia and Reperfusion Injury Effect

Total flavonoids isolated from the leaves of *C. pinnatifida* were able to reduce the degree of arrhythmia and lessen the burst size of LDH after damages in cardiocytes due to ischemia and hypoxia. Additionally, total flavonoids were able to enhance the endogenous oxygen purging system and reduce lipid peroxidation, showing it had an effect on relieving myocardial ischemia [67].

### 3.2. Digestive System Effects

#### 3.2.1. Gastrointestinal Function Regulating Effect

The alcohol extract (extracted with 60% alcohol) and the aqueous extract of *C. pinnatifida* had different effects on gastrointestinal function regulation. As for the alcohol extract, in the range of 2–8 mg/mL (crude drugs), the alcohol extract of charred fruits of *C. pinnatifida* was able to significantly reduce the contractility of rat gastric and intestine smooth muscle strips in a dose-dependent manner. [68]. In another investigation, the alcohol extract of *C. pinnatifida* could significantly reduce the contractility of rat gastric and intestine smooth muscle strips in the range of 5–20 mg/mL (crude drugs) in a dose-dependent manner, and the extract at the dose of 20 mg/mL (crude drugs) could inhibit the stimulation induced by acetylcholine [69]. For the aqueous extract, in the range of 5–20 mg crude drugs/mL, the aqueous extract of *C. pinnatifida* significantly enhanced the contractility of rat gastric and intestine smooth muscle strips in a dose-dependent manner. The extract mentioned above at the dose of 20 mg/mL could enhance the intensive contraction induced by acetylcholine and antagonize the relaxation of intestinal smooth muscle induced by atropine [70].

A common side-effect of azithromycin is gastrointestinal effects, especially for children. In a study, when injected intravenously with azithromycin, if patients (children) took hawthron slices orally, the incidence of side-effects was lower compared with the control group (*p* < 0.05). This study demonstrated that *C. pinnatifida* was able to reduce the side-effects of azithromycin on the stomach and intestine without any reported additional side-effects [71].

#### 3.2.2. Digestive Enzyme Promotion Effects

*C. pinnatifida* contains vitamin C, vitamin B2, carotene and various organic acids, which could enhance the secretion of digestive enzymes and the enzyme activity within the stomach. Especially, amylase can enhance the activity of lipase which is able to directly help to digest fatty foods, and protease agonists from *C. pinnatifida* could enhance protease activity [72]. The organic acids were able to enhance mice gastrointestinal motility, and antagonize the relaxation of intestinal smooth muscle induced by atropine, though the organic acids had no effect on the stimulation of intestinal smooth muscles induced by neostigmine. This study demonstrated that it was a one-way regulation for intestinal motility [73].

### 3.3. Effects on Pathogenic Microorganisms

#### 3.3.1. Antibacterial Effects

The antibacterial effects of *C. pinnatifida* have been comprehensively investigated. The juice squeezed from *C. pinnatifida* had antibacterial effects [74]. Additionally, the extracts of *C. pinnatifida* could inhibit various bacilli and cocci, such as *Bacteroides forsythus*, *Song bacillus*, *Smith bacillus*, *Proteusbacillus vulgaris*, *Bacillus anthraci*, *Corynebacterium diphtheria*, *Typhoid bacillus* and *Streptococcus hemolyticus*. Skin disinfectants with extract of *C. pinnatifida* fruit pit as the main germicidal ingredient had preferable sterilizing effect and stability on *Escherichia coli*, *Staphylococcus aureus*, *Pseudomonas aeruginosa* and *Candida albicans* [75].

#### 3.3.2. Synergistic Antibacterial Effects

*In vitro*, the minimal inhibitory concentrations (MICs) of oxacillin, ampicillin sodium plus sulbactam sodium, ampicillin, cephazolin, and active components of *C. pinnatifida* were 256, 512, 512, 128, and 1,024 µg/mL against methicillin-resistant *staphylococcus aureus* (MRSA), respectively. However, when combined with the active components of *C. pinnatifida* at its sub-MIC (128 µg/mL) concentration, the MICs of the above four β-lactam antibiotics were 2, 32, 16 and 2 µg/mL respectively. The results demonstrated that these active components of *C. pinnatifida* had a synergistic antibacterial effect on MRSA when combined with β-lactam antibiotics [76].

In an experiment, the bacterial strains were the standard MRSA strain WHO-2 (WHO-2) and 45 clinical MRSA strains. WHO-2 possessed a high level of resistance to oxacillin (MIC = 512 mg/L) and harbors the *mec*A gene. The 45 clinical strains were all resistant to oxacillin (MIC > 4 mg/L) and harbor the *mec*A gene. (+)-Catechin (C), (−)-epicatechin gallate (ECg) and (−)-epigallocatechin (EGC) were isolated from fructus crataegi (hawthorn) guided by antibacterial sensitization activity. The combination of (+)-catechin (C) and (−)-epicatechin gallate (ECg) could enhance the activity of β-lactam antibiotics against MRSA *in vitro* and *in vivo*, which might be related to the increased accumulation of antibiotics within MRSA via suppression of important efflux pump gene expression. It was demonstrated by two-fold dilution and checkerboard methods that C (128 mg/L) combined with ECg (16 mg/L) had the greatest effect, and the combination also reduced the bacterial load in blood of septic mice challenged with a sub-lethal dose of MRSA. The mechanism is related to increased daunomycin accumulation within MRSA and down-regulated the mRNA expression of norA, norC and abcA, three important efflux pumps of MRSA [77].

#### 3.3.3. Antiviral Effects

Human immunodeficiency virus (HIV) releases itself from an HIV-infected cell using serine protease, followed by attack on other cells. *C. pinnatifida* was found to inhibit the activity of serine protease, followed by decreasing diffusion rate of HIV *in vivo* [78]. Maslinic acid isolated from *C. pinnatifida* had a prominent effect on inhibiting the activity of HIV-1 protease. When the concentration was 17.9 mg/mL, the inhibitive rate is 100% [79]. Therefore, maslinic acid is considered as a potential candidate for novel anti-HIV therapeutics.

### 3.4. Effects on Tumors and the Immune System

#### 3.4.1. Anticancer and Sperm Distortion Inhibiting Effects

The anticancer effect of maslinic acid was investigated in 1989. Maslinic acid isolated from *C. pinnatifida* exhibited cytotoxicity on P-38 cancer cells (ED_50_ = 13.0 µg/mL) [80]. Total flavonoids isolated from *C. pinnatifida* had no effect on normal cells, but could obviously enhance calcium concentration in tumor cells. *In vitro*, total flavonoids inhibited and killed Hep-2 tumor cells by calcium overload, as well as inhibited DNA biosynthesis of tumor cells [81]. Aqueous extracts of *C. pinnatifida* were found to inhibit sperm distortion of mice induced by cyclophosphamide, which obviously reduced the number of distorted sperm. The mechanism was related to the abundant linoleic acid and vitamin C in *C. pinnatifida* [82].

#### 3.4.2. Immunoregulating Effects

In an experiment, the 100% decoction of *C. pinnatifida* was used in mice (*p.o.*, 0.2 mL/10 mg/d, for 9 days). It was shown that the decoction could increase the weight of the immune organs of the mice (thymus and spleen), raise T lymphocyte transformation rate and T lymphocyte ANAE cell percentage, indicating the decoctum of *C. pinnatifida* has an obvious improving effect on the cellular immune function of the mice, which also provides an experimental basis for the clinical application [83]. The injection of *C. pinnatifida* (water extract and alcohol precipitate, 1 g crude drugs/mL) had the same effect as the decoction [84]. Sitosterol isolated from *C. pinnatifida* was able to significantly increase the leucocyte count and enhance the phagocytic activity of macrophages, and had effects on spleen and lymphocytes in mice model of immunosuppression induced by cyclophosphamide (CTX) [85]. It was confirmed that the polysaccharides extracted from *C. pinnatifida* were able to enhance the spleen, thymus and the phagocytic activity of macrophages, promote the formation of hemolysin and hemolysis plaque of mice [86].

### 3.5. Endocrine System Effects

Endocrine system imbalance is a major factor of diabetes. The regulation of the hepatic glucose output through glycogenolysis is an important target for type II diabetes therapy. Glycogenolysis is catalyzed in liver, muscle and brain by tissue specific isoforms of glycogen phosphorylase (GP). Because of its central role in glycogen metabolism, GP had been exploited as a model for structure assisted design of potent inhibitors, which might be relevant to the control of blood glucose concentrations in type II diabetes [87,88]. Maslinic acid isolated from *C. pinnatifida* was found to inhibit GP in moderate strength; the IC50 was 28 µmol/L. As glycogen phosphorylase inhibitors, the best effect of maslinic acid derivatives was 4 times better than maslinic acid [89]. Maslinic acid (10 µg/kg/day or 30 µg/kg/day, two weeks) was found to obviously lessen the levels of blood glucose in KK-Ay mice by lessening insulin resistance of KK-Ay mice, these results suggested that maslinic acid might be investigatedas a new drug for type II diabetes treatment [90].

### 3.6. Coagulation System Effects

*In vitro*, the extracts from the leaves of *C. pinnatifida* were reported to inhibit platelet aggregation of rabbit [91]. In another study, the IC50 of *C. pinnatifida* on platelet aggregation induced by ADP was 1.388% (g crude drugs per 100mL) [92]. In acute blood stasis rat model, total flavonoids obviously influenced the hemorheology, which decreased viscosity of plasma and hematocrit [93]. Total flavonoids isolated from the leaves of *C. pinnatifida* were found to inhibit thrombogenesis caused by vascular endothelial injury of artery. The mechanism is related to the enhancement of the surface charge and speeding up the fluxion of erythrocyte and soterocyte, lessening the gather and adhesion [94,95]. At low doses (between 100–500 mg/kg), *C. pinnatifida* water extracts was reported to inhibit platelet function significantly in Wistar albino rats. The extracts were able to change the bleeding time and the closure time, which determined by the PFA-100 and thromboxane B2 levels [96].

### 3.7. Other Effects

#### 3.7.1. Antiinflammatory Effects

The inhibitory effect of ethanol extract from the leaves of *C. pinnatifida* on mice ear inflammation induced by dimethylbenzene was investigated [97,98]. The results showed ethanol extract of *C. pinnatifida* has definite antiinflammatory effects.

#### 3.7.2. Antioxidant Effects

Total flavonoids of *C. pinnatifida* leaves was found to have a strong ability to scavenge oxygen free radicals, enhance superoxide dismutase (SOD) activities, and lessen malondialdehyde (MDA) levels. This result demonstrated that total flavonoids of *C. pinnatifida* leaves had good effects on protecting brain tissue, nephridial tissue, hepatic tissue and neuron by remitting oxidative stress [99,100,101,102].

#### 3.7.3. Osteoporosis Inhibiting Effects

In a simulative animal model of osteoporosis induced by menopause, maslinic acid isolated from *C. pinnatifida* was found to inhibit osteoporosis. The mechanism was that maslinic acid was able to inhibit downstream-signal (NFκB) activities and transcription-factor (NFATcl) expressions, but it had no effects on transcriptional activities of NFATcl. Additionally, maslinic acid could also regulate the downstream-signalling (MAPK); but it had no effects on calcium flow oscillation. Therefore, the results suggested maslinic acid might be used as a new drug against osteoporosis induced by menopause [103].

#### 3.7.4. Retina Protecting Effects

In an experiment, experimental rabbits were contaminated by inhaling CS_2_ for 3 continuous hours on 6 consecutive days a week for a total of 3 weeks. The rabbits in the treatment group were given “haw drink compound” (water decoction with *Crataegus pinnatifida*, *Lycium barbarum*, *Fructus jujubae*) before contamination. After 3 weeks of the experiment, the results showed that the ultrastructures of the retinal tissues of the control group were more abnormal than those of the treatment group and the normal group. Every layer cell of the retinal in the control group showed apparent degenerative changes, but that in the treatment group was normal. This investigation demonstrated that haw drink compound could improve the tolerance to CS_2_ toxicity in inducing the retinal damage of rabbits [104].

## 4. Toxicology

*C. pinnatifida* has been used for hundreds of years as an important traditional herbal medicine in China, as well as a daily foodstuff. However, studies of the relative systematic toxicity and safety of *C. pinnatifida* are lacking. So far, oral corn pollen haw liquor (an oral solution containing corn, pollen and haw) was demonstrated no have no genotoxicity effects [5]. Additionally, in order to ensure the safety of drug use, acute and long-term toxicity experiments with “semen cassiae hawthorn oat” (a capsule contains semen cassia, hawthorn and oat) were investigated. For the acute toxicity reactions, the semen cassiae hawthorn oat was orally given one-time at 10 g/kg dosages (the maximum tolerated dose in mice), and observed continuously for 14 days. For the long-term toxicity reactions, the semen cassiae hawthorn oat was orally given continuously for 8 weeks at low (1.6 g/kg) and high (2 g/kg) dosages. In the acute toxicity experiments, there were no toxic reactions or animal deaths. In the long-term toxicity experiments, there was no significant differences in the general state, weight changes, hematological indexes, biochemical indexes and organ coefficients in rats of low and high dosage group when compared with the control group. Pathologic examination did not show any structural and cellular abnormalities of each organ [105]. The above results demonstrated that *C. pinnatifida* is safe and non-toxic for experimental animals.

## 5. Future Perspectives and Conclusions

Medicinal plants are universally considered as important sources of new chemical substances with potential therapeutic effects. *C. pinnatifida* has long been used in Traditional Chinese Medicine for the treatment of cardiovascular disease, dyspepsia, infections and cancers. The flavonoids are considered to be the major bioactive constituents. *C. pinnatifida* has been of increasing interest in recent years, and many traditional uses have been investigated, but there is not enough systemic data about the toxicity and safety of *C. pinnatifida*, and few target-organ toxicity evaluations have been documented. Therefore, more investigations should be done regarding the toxicity and pharmacokinetics of *C. pinnatifida*.

In Traditional Chinese Medicine, *C. pinnatifida* is commonly used in compositions with other herbs and not used alone. Although many of the experimental results validate that *C. pinnatifida* exhibits significant pharmacological effects when used alone, it’s important to investigate the pharmacological effects and molecular mechanisms of *C. pinnatifida* combined with other herbs based on modern concepts of disease pathophysiology. Furthermore, drug target-guided and bioactivity-guided isolation and purification of the chemical constituents and subsequent evaluation of the pharmacologic effects will promote the development of bioactive constituents and expand our knowledge of *C. pinnatifida*. Detailed investigations of the pharmacology, molecular mechanisms of action and systems biology will help to ensure which chemical constituents or multiple ingredients contribute to its pharmacological effects and help develop new effective drugs which could produce enormous benefit to society and the economy.
